# Prognostic scores and biomarkers in hematologic malignancies: current evidence and future perspectives

**DOI:** 10.1186/s40364-026-01033-8

**Published:** 2026-10-31

**Authors:** Eliot McDonald, Emanuela Marcenaro, Philippe Robert, Pascal Rihet, Olivier Théodoly, Régis Costello

**Affiliations:** 1 Aix Marseille University, TAGC/INSERM UMR1090, Parc Scientifique de Luminy, Marseille, France; 2 Aix Marseille University, INSERM, CNRS, Laboratoire Adhésion et Inflammation (LAI), UMR 7333, U1067, Parc Scientifique de Luminy, Marseille, France; 3 Department of Experimental Medicine, University of Genova, Genova, Italy; 4 AOM - IRCCS Ospedale Policlinico San Martino, Genova, Italy; 5 Assistance Publique Hôpitaux de Marseille, Laboratoire d’Immunologie – Hôpital de la Timone, Marseille, 13005 France; 6 Assistance Publique Hôpitaux de Marseille, Service Hématologie & Thérapie Cellulaire– Hôpital de la Conception, Marseille, 13005 France

**Keywords:** Immunotherapy, Hematologic malignancies, Prognostic scores, CAR- T cells, Bispecific antibodies, Immune checkpoint inhibitors, Inflammation, Cytokines, Predictive biomarkers

## Abstract

The advent of immunotherapy has profoundly transformed the therapeutic landscape of hematologic malignancies. A broad range of immune-based strategies, including immune checkpoint inhibitors and adoptive cell therapies based on both T cells and natural killer (NK) cells, have achieved unprecedented clinical outcomes in several lymphoid and myeloid neoplasms. Emerging approaches such as genetically engineered immune cells, including chimeric antigen receptor (CAR)-modified T and NK cells, are further expanding therapeutic opportunities. However, substantial heterogeneity in treatment response remains a major challenge, with a significant proportion of patients experiencing primary resistance, relapse, or severe immune-related toxicities. Accurate identification of predictive and prognostic biomarkers is therefore essential to optimize patient selection, improve therapeutic efficacy, and minimize treatment-related toxicity. Numerous biological parameters have been investigated, including clinical prognostic scores, systemic inflammatory indices, tumor burden markers, immune-cell subsets, cytokine profiles, genetic and molecular alterations, and features of the tumor microenvironment. Emerging evidence indicates that both host-related factors and tumor-intrinsic characteristics shape therapeutic responses to immunotherapy across hematologic cancers. Among the most promising biomarkers are inflammatory indices derived from peripheral blood counts, such as the neutrophil-to-lymphocyte ratio, platelet-to-lymphocyte ratio, monocyte-to-lymphocyte ratio, systemic immune-inflammation index, prognostic nutritional index, and Glasgow Prognostic Score. In parallel, immune-monitoring approaches assessing the functional competence, phenotype, and activation status of both T and NK cells, together with cytokine signatures, immune checkpoint expression, and other markers of immune competence, are increasingly recognized as valuable tools for predicting therapeutic response, resistance, and immune-related toxicity. This review summarizes the current evidence on prognostic scores and biomarkers associated with immunotherapy outcomes in hematologic malignancies. We discuss their biological rationale, clinical relevance, current limitations, and potential integration into contemporary treatment strategies. Finally, we highlight emerging perspectives, including multi-omics profiling, artificial intelligence-driven predictive models, and dynamic biomarker-guided approaches, which are expected to advance precision immuno-oncology and enable increasingly personalized immune-based treatment algorithms.

**Graphical Abstract:**

**Figure:**
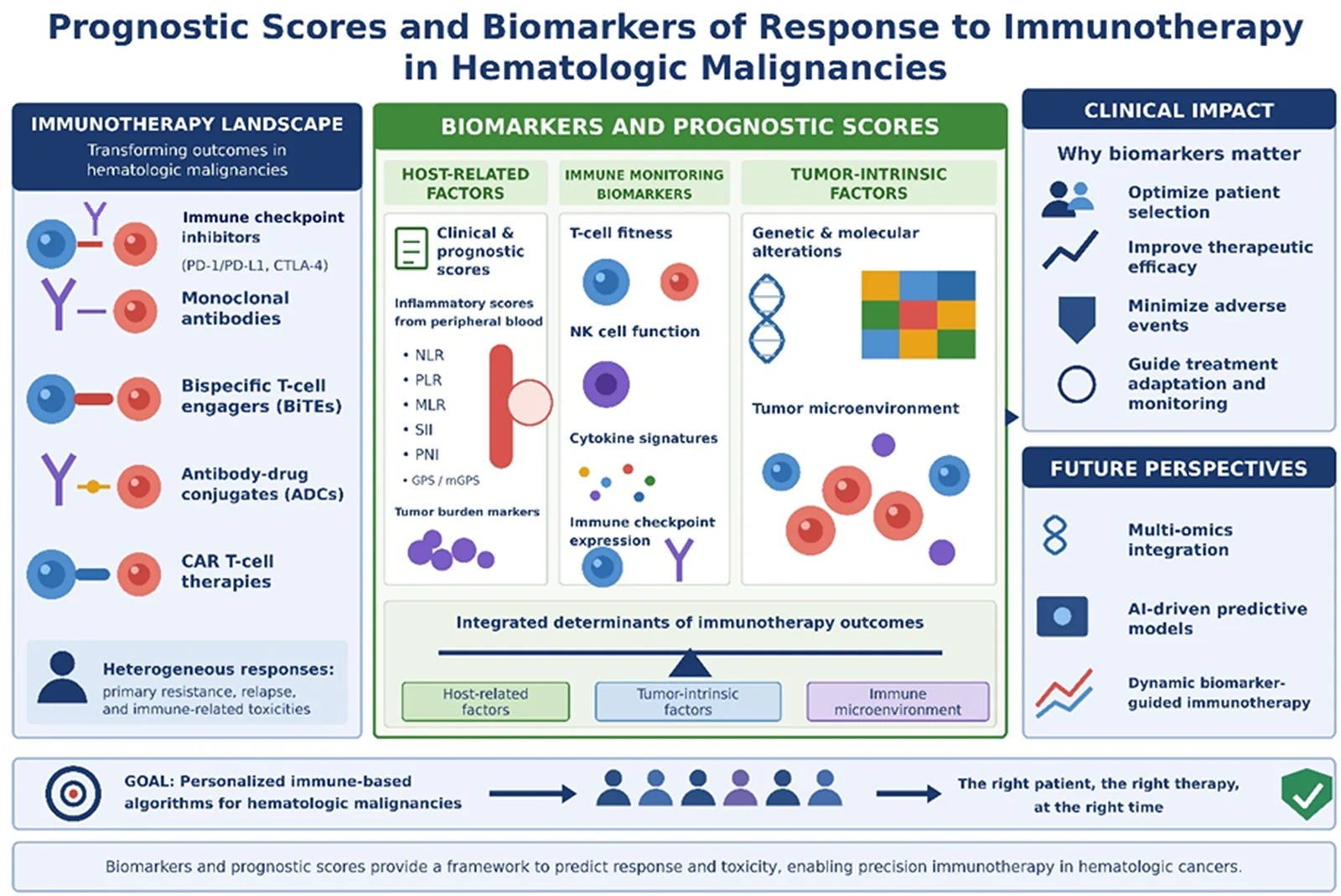

**Supplementary Information:**

The online version contains supplementary material available at https://doi.org/10.1186/s40364-026-01033-8.

## Introduction

The treatment of hematologic malignancies has undergone a remarkable transformation over the past decade with the emergence of immunotherapeutic approaches. Building upon the success of allogeneic hematopoietic stem cell transplantation and monoclonal antibody-based therapies, novel immune-based strategies have significantly improved outcomes in patients with lymphoid and myeloid neoplasms. These advances include immune checkpoint inhibitors [1] antibody-based therapies, and adoptive cellular immunotherapies based on genetically engineered T and NK cells [2–4]. Several of these modalities have achieved durable remissions in heavily pretreated patients who previously had limited therapeutic options.

Despite these advances, response to immunotherapy remains highly variable. While some patients experience long-term disease control or even cure, others exhibit primary resistance, fail to achieve meaningful responses, or rapidly relapse following an initial response. Furthermore, immune-based therapies can be associated with potentially life-threatening toxicities, including cytokine release syndrome (CRS), immune effector cell-associated neurotoxicity syndrome (ICANS), prolonged cytopenia, infections, and immune-related adverse events. This considerable interindividual variability highlights the urgent need for reliable biomarkers capable of predicting treatment efficacy and toxicity before therapy initiation. These determinants are likely to differ across immunotherapeutic modalities, reflecting their distinct mechanisms of action and immune effector populations.

They involve interactions between tumor-intrinsic characteristics, host immune competence, systemic inflammation, treatment-related variables, and the tumor microenvironment. In contrast to solid tumors, where biomarkers such as PD-L1 expression, microsatellite instability, and tumor mutational burden have been extensively investigated, predictive biomarkers in hematologic malignancies remain less clearly established [5]. The biological heterogeneity of hematologic cancers, coupled with their unique interactions with the immune system, presents additional challenges for biomarker development and validation. Although numerous biomarkers have been investigated within individual therapeutic settings, few have demonstrated consistent predictive value across different immunotherapeutic modalities, highlighting the need for more comprehensive and integrative biomarker strategies.

Recent research has identified several categories of potentially informative biomarkers. Tumor-related factors include antigen expression density, molecular and cytogenetic abnormalities, tumor burden, clonal evolution, and mechanisms of immune escape. Host-related parameters encompass age, performance status, nutritional status, systemic inflammation, immune-cell composition and functional immune-competence. Particular attention has recently focused on peripheral blood-derived inflammatory scores, which are inexpensive, reproducible, and readily available in routine clinical practice. These include the neutrophil-to-lymphocyte ratio (NLR), platelet-to-lymphocyte ratio (PLR), lymphocyte-to-monocyte ratio (LMR), monocyte-to-lymphocyte ratio (MLR), systemic immune-inflammation index (SII), prognostic nutritional index (PNI), and Glasgow Prognostic Score (GPS/mGPS). Such markers have demonstrated prognostic significance across multiple malignancies and are increasingly being evaluated as predictors of response to immunotherapeutic interventions.

Beyond conventional hematological parameters, advances in immune monitoring technologies have enabled a more comprehensive characterization of the immune landscape. High-dimensional flow cytometry, mass cytometry, single-cell RNA sequencing, spatial transcriptomics, and multiplex cytokine profiling have revealed intricate relationships between immune-cell subsets and therapeutic outcomes. Functional and phenotypic characteristics of T cells and NK cells, together with the abundance and activation state of myeloid-derived suppressor cells, regulatory T cells, macrophages, and other components of the tumor immune microenvironment, have emerged as important determinants of immunotherapy efficacy, resistance, and treatment-related toxicity. Similarly, circulating cytokines such as interleukin-6 (IL-6), interleukin-10 (IL-10), interferon-γ (IFN-γ), tumor necrosis factor-α (TNF-α), and soluble immune checkpoints have demonstrated prognostic and predictive potential in several settings, particularly in patients receiving CAR-T cell therapies and other adoptive cellular immunotherapies.

As immunotherapy continues to expand across hematologic malignancies, there is increasing interest in integrating multiple biomarkers including clinical, inflammatory, immunological, molecular, and spatial biomarkers, into composite predictive models. Such approaches may capture the dynamic interactions between tumor biology and host immunity than individual markers alone. The emergence of machine learning algorithms and multi-omics platforms further raises the possibility of developing highly personalized predictive tools capable of guiding treatment selection and monitoring.

In this review, we examine the current evidence regarding prognostic scores and biomarkers associated with immunotherapy outcomes in hematologic malignancies. We discuss inflammatory indices, immune-cell population’s functional states, cytokine signatures, molecular and genetic biomarkers, as well as emerging composite predictive models across different immunotherapeutic modalities. We also address current limitations and future directions toward the implementation of biomarker-driven precision immuno-oncology in hematology.

A structured literature search was performed to identify clinical, translational, and biological studies addressing prognostic and predictive biomarkers and scoring systems in patients with hematologic malignancies receiving immunotherapy. **PubMed/MEDLINE** was used as the primary bibliographic database. Additional relevant publications were identified by screening the reference lists of key articles and recent reviews and by backward and forward citation tracking of highly relevant publications.

The search covered publications from **January 2010 to September 2026**, with particular emphasis on studies published between **January 2021 and September 2026**, reflecting the rapid expansion of contemporary immunotherapeutic approaches and biomarker research. Earlier publications were retained when they represented seminal studies establishing the development, validation, or biological rationale of a prognostic score or biomarker subsequently investigated in the immunotherapy setting.

The search strategy combined terms related to the **disease setting**, **therapeutic modality**, and **biomarker or outcome**. Core search concepts included combinations of the following terms: *“hematologic malignancies*,*” “hematological malignancies*,*” “acute leukemia*,*” “acute myeloid leukemia*,*” “lymphoma*,*” “multiple myeloma*,*” “chronic lymphocytic leukemia*,*” “myelodysplastic syndrome*,*” “immunotherapy*,*” “immune checkpoint inhibitor*,*” “CAR-T*,*” “CAR T-cell*,*” “bispecific antibody*,*” “T-cell engager*,*” “monoclonal antibody*,*” “cellular therapy*,*” “biomarker*,*” “prognostic score*,*” “predictive biomarker*,*” “inflammatory score*,*” “immune signature*,*” “immune-cell fitness*,*” “T-cell*,*” “NK-cell*,*” “cytokine*,*” “EASIX*,*” “CAR-HEMATOTOX*,*” “tumor burden*,*” “MRD*,*” “ctDNA*,*” “single-cell*,*” “transcriptomic*,*” “functional assay*,*”* and *“immune-related toxicity.”* These terms were combined using Boolean operators (AND/OR) and adapted as appropriate to the terminology of the database.

Studies were considered relevant when they evaluated one or more of the following: (i) established clinical prognostic scores; (ii) systemic inflammatory or nutritional indices, including NLR, PLR, MLR, LMR, SII, PNI, and GPS/mGPS; (iii) endothelial biomarkers, including EASIX and mEASIX; (iv) CAR-T-specific scores, including CAR-HEMATOTOX and related models; (v) T-cell and NK-cell phenotype, fitness, differentiation, exhaustion, or functional competence; (vi) cytokine and inflammatory signatures; (vii) molecular, genetic, transcriptomic, or single-cell biomarkers; (viii) circulating tumor DNA and measurable residual disease; and (ix) composite, multimodal, or artificial-intelligence-based predictive models. Particular attention was given to studies reporting associations with treatment response, complete remission, progression-free survival, overall survival, relapse, cytokine release syndrome (CRS), immune effector cell-associated neurotoxicity syndrome (ICANS), prolonged cytopenias, infections, non-relapse mortality, or other clinically relevant treatment-related outcomes.

Studies were generally excluded when they did not concern hematologic malignancies, did not involve an immune-based therapeutic approach, or did not provide clinically or biologically relevant prognostic or predictive information. Studies focused exclusively on conventional chemotherapy or targeted therapies were not included in the main evidence synthesis unless they represented seminal publications establishing the original development or biological rationale of a prognostic score or biomarker subsequently evaluated in the immunotherapy setting. Case reports and very small case series were generally excluded from the main evidence synthesis, whereas methodological or conceptual publications were retained when they provided essential context for emerging biomarker approaches.

Because the present manuscript was designed as a **narrative review rather than a systematic review or meta-analysis**, the search strategy was intended to provide broad and clinically relevant coverage of an evolving field rather than to constitute an exhaustive PRISMA-compliant systematic search. The level of evidence and degree of clinical validation of individual biomarkers were therefore considered when interpreting the available literature, with a distinction made between established prognostic associations, emerging biomarkers, and biomarkers with demonstrated predictive potential.

## Classical clinical prognostic scores as predictors of immunotherapy outcomes in hematologic malignancies (Table 1)

Before the advent of immunotherapy, prognostic assessment in hematologic malignancies relied primarily on clinical and disease-specific scoring systems developed to estimate survival, disease progression, and treatment response. These classical prognostic models incorporate readily available clinical, biological, and cytogenetic variables and remain central to therapeutic decision-making in routine practice. Although originally designed for conventional chemotherapy, immunochemotherapy, or transplantation settings, increasing evidence suggests that these scores also retain prognostic significance in patients treated with modern immunotherapeutic approaches.

The relevance of classical prognostic scores in immunotherapy stems from the fact that many variables included in these models reflect fundamental determinants of immune competence and disease biology, such as tumor burden, performance status, age, bone marrow reserve, inflammatory status, and genetic risk. Consequently, these scores may indirectly influence both treatment efficacy and toxicity following immune-based therapies.

Several studies have demonstrated that established prognostic indices continue to stratify outcomes in patients receiving immune checkpoint inhibitors, bispecific antibodies, antibody-drug conjugates, and CAR-T cell therapies. Nevertheless, the predictive value of these tools remains imperfect because they were not specifically designed to capture immune-related mechanisms. Their integration with emerging immunological biomarkers therefore represents a major area of current research.

### Prognostic scores in lymphoid malignancies

The International Prognostic Index (IPI), developed by the International Non-Hodgkin’s Lymphoma Prognostic Factors Project in 1993, remains one of the most widely used prognostic tools in aggressive B-cell lymphomas [6]. The IPI incorporates five adverse prognostic factors: Age > 60 years, Elevated serum lactate dehydrogenase (LDH), Eastern Cooperative Oncology Group (ECOG) performance status ≥ 2, Ann Arbor stage III-IV, more than one extranodal disease site. Patients are classified into four risk categories associated with significantly different survival outcomes. In the era of CAR-T cell therapy, several studies have shown that a high IPI remains associated with inferior progression-free survival (PFS) and overall survival (OS). For example, analyses of patients receiving anti-CD19 CAR-T cells demonstrated that elevated IPI scores correlate with increased tumor burden, reduced CAR-T expansion, and higher relapse rates [7, 8]. Similarly, in patients treated with bispecific antibodies such as epcoritamab or glofitamab [9], historical clinical risk factors, including components of the IPI, remain associated with outcomes, suggesting that disease aggressiveness remains a determinant of immunotherapy response.

The R-IPI was developed following the incorporation of rituximab into lymphoma treatment [10]. This simplified model divides patients into very good risk, good risk and poor risk. The R-IPI has also demonstrated utility in identifying patients with diffuse large B-cell lymphoma (DLBCL) who derive durable benefit from CAR-T therapy. Real-world studies have reported mixed and sometimes contradictory findings regarding the prognostic value of the R-IPI in patients receiving CAR-T-cell therapy, with some cohorts associating poor-risk R-IPI categories with lower complete remission rates and shorter survival, whereas others have not confirmed a significant association. Thus, the R-IPI may be associated with CAR-T efficacy in selected cohorts, but its predictive and prognostic value remains inconsistent across studies [11–13]. Recognizing the limitations of the original IPI, the National Comprehensive Cancer Network developed the NCCN-IPI [14]. This score refines risk stratification by incorporating detailed age categories, LDH level stratification, and specific extra nodal disease sites. Among patients receiving contemporary cellular therapies, the NCCN-IPI has shown superior prognostic performance compared with the traditional IPI. Several retrospective studies have demonstrated that higher IPI-derived scores, including the NCCN-IPI, retain prognostic value in the CAR-T setting and are associated with inferior PFS and OS in patients with relapsed/refractory DLBCL. The Follicular Lymphoma International Prognostic Index (FLIPI) [15] was established for follicular lymphoma and includes five variables: age > 60 years, Ann Arbor stage III-IV, hemoglobin < 12 g/dL, elevated LDH, more than four nodal areas. Designed for chemoimmunotherapy-treated patients, the FLIPI does not predict outcomes in patients receiving modern immunotherapeutic approaches treatment by CAR-T cells [16, 17], although patients with high FLIPI scores frequently exhibit increased disease burden and a more immunosuppressive tumor microenvironment, factors associated with reduced immune-mediated tumor eradication.

The Chronic Lymphocytic Leukemia International Prognostic Index [18] (CLL-IPI) combines clinical and biological parameters: age, clinical stage, β2-microglobulin, TP53 status, IGHV mutational status. While targeted therapies have modified the natural history of CLL, the CLL-IPI remains predictive of outcomes. Emerging studies evaluating CAR-T therapies and bispecific antibodies suggest that high-risk CLL-IPI patients demonstrate lower response durability and shorter survival, likely reflecting adverse biological features rather than immunotherapy resistance alone, but this score is not the most relevant for prognosis [19] instead of the more relevant numeration of memory-like T -cells, transcriptional study of T-cells and presence of an exhausted phenotype.

### Prognostic scores in multiple myeloma

For multiple myeloma (MM), the International Staging System (ISS) is based on measuring serum β2-microglobulin and serum albumin [20]. Despite its simplicity, the ISS remains predictive in patients receiving B-cell maturation antigen (BCMA)-targeted therapies. Patients with ISS stage III generally experience higher tumor burden, lower response durability, and inferior progression-free survival following CAR-T therapy. This association has been observed in studies evaluating idecabtagene vicleucel and ciltacabtagene autoleucel. Nonetheless, in the paper of Munshi et al. [21] the authors do not provide separate Kaplan-Meier curves or median PFS/OS according to ISS stage but state that clinical activity was observed across high-risk subgroups including patients with high tumor burden, high-risk cytogenetics, extramedullary disease and triple-class refractory disease; also, the ISS does not really seem to be a strong predictor of response. Similar conclusions were reached by the study of Berdeja et al. [22] The Revised International Staging System (R-ISS) and the R2-ISS incorporates ISS stage, high-risk cytogenetics and LDH levels [23]. Because the R-ISS incorporates high-risk cytogenetic abnormalities and elevated LDH, both of which have repeatedly been associated with inferior outcomes after BCMA-directed CAR-T therapy, it is plausible that the R-ISS retains prognostic value in this setting. A distinction must be made between a prognostic factor and a validated prognostic score. The R-ISS score was developed in patients treated with conventional therapies and has not been formally validated as a CAR-T-specific prognostic model. Likewise, the recently developed R2-ISS, which incorporates 1q abnormalities, may provide improved risk stratification, but prospective validation in CAR-T-treated cohorts is still lacking. Current evidence suggests that integrating classical staging systems with immune-related and treatment-specific biomarkers will likely yield superior prognostic models. The R-ISS and R2-ISS should not be viewed as obsolete in the CAR-T era. Rather, they should constitute the clinical backbone upon which CAR-T-specific variables (tumor burden, extra medullary disease, T-cell fitness, inflammatory biomarkers, measurable residual disease [MRD], and CAR-T expansion) can be added to create next-generation prognostic scores [24].

### Prognostic scores in myelodysplastic syndromes

The original International Prognostic Scoring System (IPSS) for myelodysplastic syndromes (MDS) incorporates bone marrow percentage of blast cells, cytogenetic abnormalities, number of cytopenias [25]. Although developed before immunotherapy became available, the IPSS remains relevant in studies evaluating immune checkpoint inhibitors, anti-CD47 therapies, and cellular therapies in MDS. Higher-risk IPSS categories are generally associated with lower response rates and reduced survival.

The revised IPSS (IPSS-R) provides refined risk stratification through expanded cytogenetic classification, deeper cytopenia assessment, and more precise blastic cell categorization [26]. Recent studies investigating magrolimab-based regimens and immune checkpoint inhibitors in MDS continue to use IPSS-R as the reference prognostic model [27]. Patients with very high-risk IPSS-R scores demonstrate poorer outcomes regardless of immunotherapy administration, but current evidence does not support R-IPSS use as a predictive biomarker for response to CD47 blockade. Rather, magrolimab studies have focused on molecular features, particularly TP53 mutation status and adverse cytogenetics, which appear more biologically relevant for treatment response.

The molecular IPSS (IPSS-M) integrates molecular genetics with traditional prognostic variables [28]. The score incorporates mutations affecting TP53, ASXL1, RUNX1, EZH2, SF3B1and other recurrent genes. IPSS-M currently represents the most comprehensive prognostic tool for MDS and may become increasingly relevant for selecting patients eligible for future immunotherapeutic interventions. Could the IPSS-M nevertheless prove useful for immunotherapy? The IPSS-M incorporates 31 genes involved in the biology of MDS. Several of these alterations are likely to influence inflammation in the microenvironment, antigen presentation, immune evasion, and sensitivity to macrophages and T cells. Consequently, it is plausible that the IPSS-M indirectly captures certain determinants of the immune response. However, this hypothesis has not yet been clinically demonstrated and requires dedicated prospective studies.

Allogeneic hematopoietic stem cell transplantation (allo-HSCT) remains the only potentially curative treatment for higher-risk MDS, and decision-analysis models as well as prospective donor-versus-no-donor studies support a survival benefit of an early transplant strategy, including in patients aged 50 to 75 years treated with reduced-intensity conditioning (RIC) [29–32]. The BMT CTN 1102 trial reported better leukemia-free survival at 3 years in patients with a matched donor than in those without one, and the VidazaAllo study found that RIC transplantation after azacitidine induction improved event-free survival compared with continuous azacitidine in patients aged 55 to 70 years. Outcome after transplantation is nevertheless shaped by the interplay of patient-, disease- and transplant-related variables. Non-relapse mortality (NRM) is driven mainly by comorbidities, as captured by the HCT-specific comorbidity index (HCT-CI) [33]. In a large EBMT registry analysis of 1,333 MDS patients aged 50 years or older, advanced disease stage at transplantation was the major independent factor associated with inferior OS, whereas recipient age and conditioning intensity were not [34]. Disease-related factors determine the risk of relapse, which is the leading cause of treatment failure: these include the IPSS-R risk category [26], and above all cytogenetics, with complex and monosomal karyotypes conferring markedly inferior survival after transplantation; the Disease Risk Index integrates disease type, stage and cytogenetics into a transplant-specific risk score [35]. Molecular profiling has refined this picture: *TP53* mutations, and mutations of RAS pathway genes and *JAK2*, are associated with early relapse and shorter survival after allo-HSCT, and several cohorts have confirmed the adverse impact of *TP53* and other driver mutations [36–38]. The molecular IPSS (IPSS-M) now incorporates genomic data into diagnostic risk assessment [28]. The intensity of conditioning trades relapse against toxicity. In the randomized BMT CTN 0901 trial in AML and MDS, RIC was associated with a much higher relapse incidence than myeloablative conditioning (48.3% versus 13.5%), but with lower treatment-related mortality (4.4% versus 15.8%), and the authors concluded that myeloablative conditioning should remain the standard for fit patients [39]. In contrast, the EBMT RICMAC trial, which enrolled 129 patients with MDS or secondary AML, found that RIC gave RFS and OS at 2 years at least similar to myeloablative conditioning [40]. Conditioning should therefore be chosen according to fitness, comorbidity burden and disease risk rather than age alone. Finally, hypomethylating agents are widely used as pre-transplant cytoreduction or as a bridge while a donor is identified, as in VidazaAllo [31], but their benefit on post-transplant outcome compared with upfront transplantation has not been established in randomized trials.

### Prognostic scores in Hodgkin lymphoma

The International Prognostic Score (IPS) was developed for advanced-stage classical Hodgkin lymphoma and includes seven adverse variables [41]. Several studies evaluating nivolumab and pembrolizumab have shown their clinical interest without firmly demonstrating correlation with the IPS [42, 43]. The study of Chen et al. [43] in addition to standard scores reported three other scores: the PD-L1 staining intensity (0–3), the membrane staining score (% of tumor cell expressing PD-L1, [0–3]) and histiocyte score, i.e. the semiquantitative assessment of histiocyte/macrophages staining positivity for PD-L1 (0–3). Each independent score was associated to a better prognosis (more particularly from 0 to 1 to 2–3, but 64.4% of patients had the maximum score across all three PD-L1 expression scores (intensity = 3, histiocyte = 3, tumor staining = 3) so that the prognosis/predictive values remain difficult to be firmly confirmed (114 patients for a total of 177 patients including 90.4% with highest intensity staining, 88.1% with 100% PD-L1 positive membrane staining, and 71.8% with maximum histiocyte staining).

**Table 1:** Major classical clinical prognostic scores used in hematologic malignancies

Score	Disease	Main Variables	Clinical Use	Relevance in Immunotherapy
IPI	DLBCL	Age, LDH, ECOG, stage, extranodal sites	Aggressive lymphoma prognosis	Predicts CAR-T and bispecific antibody outcomes
R-IPI	DLBCL	Simplified IPI categories	Survival stratification	Associated with CAR-T efficacy
NCCN-IPI	DLBCL	Refined age, LDH, extranodal involvement	Improved risk discrimination	Superior prognostic performance in CAR-T cohorts
FLIPI	Follicular lymphoma	Age, stage, Hb, LDH, nodal sites	Risk stratification	Prognostic value after CAR-T and bispecific therapies not proven
CLL-IPI	CLL	Age, stage, β2M, TP53, IGHV	Survival prediction	May identify high-risk patients for cellular therapies
ISS	Multiple myeloma	β2M, albumin	Disease staging	Predicts CAR-T response durability
R-ISS	Multiple myeloma	ISS + cytogenetics + LDH	Refined prognosis	Associated with PFS after BCMA-targeted therapies
IPSS	MDS	blasts counts, cytopenias, cytogenetics	MDS risk assessment	Baseline prognostic tool in immunotherapy studies
IPSS-R	MDS	Refined cytogenetics and cytopenias	Current clinical standard	Widely used in immune-based clinical trials
IPSS-M	MDS	IPSS-R + molecular mutations	Precision prognostication	Emerging role in patient selection
IPS	Hodgkin lymphoma	Seven clinical variables	Advanced-stage prognosis	Associated with PD-1 inhibitor outcomes

Classical clinical prognostic scores remain highly relevant in the immunotherapy era despite being developed before the emergence of modern immune-based treatments. Across lymphoid and myeloid malignancies, these scores continue to capture essential determinants of treatment outcome, including disease burden, biological aggressiveness, patient fitness, and hematopoietic reserve. However, their predictive capacity remains limited because they do not directly evaluate immune competence, tumor microenvironment composition, immune exhaustion, or inflammatory status

## Inflammatory prognostic scores (Table 2)

Cancer-related inflammation is increasingly recognized as a hallmark of tumor progression and immune escape. Beyond its role in oncogenesis, systemic inflammation profoundly influences the efficacy of anti-tumor immune responses and has emerged as a major determinant of outcomes following immunotherapy. In hematologic malignancies, inflammatory biomarkers derived from routine blood counts and biochemical parameters have attracted growing interest because they provide a simple, inexpensive, and reproducible assessment of the host immune status. The rationale behind inflammatory prognostic scores lies in the balance between protumoral inflammatory processes and antitumor immune surveillance. Elevated neutrophils, monocytes, and platelets may promote tumor growth through cytokine secretion, angiogenesis, and suppression of cytotoxic lymphocyte activity, whereas lymphocytes represent the cornerstone of adaptive antitumor immunity [44, 45]. Several composite inflammatory indices have therefore been developed, including NLR, PLR, MLR, LMR, SII, PNI and GPS/mGPS. These markers have demonstrated prognostic significance across multiple hematologic malignancies and are increasingly investigated as predictors of response to immune-based therapies.

### Neutrophil-to-lymphocyte ratio

The neutrophil-to-lymphocyte ratio is calculated as **NLR = Absolute Neutrophil Count / Absolute Lymphocyte Count.** Among inflammatory biomarkers, NLR is the most extensively studied in oncology [46]. Elevated neutrophil counts are associated with increased production of vascular endothelial growth factor (VEGF), transforming growth factor-beta (TGF-β), interleukin-6 (IL-6), and reactive oxygen species, all of which contribute to tumor progression and immune suppression [47]. Conversely, lymphopenia reflects impaired cellular immunity and diminished antitumor surveillance. In solid tumors, a high baseline NLR consistently predicts inferior response rates and survival [48], including treatment by immune checkpoint blockade such as in non-small cell lung cancer [49] and advanced melanoma [50] treated by nivolumab. Baseline neutrophil-to-lymphocyte ratio (NLR) is a well-established prognostic biomarker in classical Hodgkin lymphoma. Large multicenter studies demonstrated that a NLR > 6 independently predicts inferior PFS and OS. However, despite the widespread use of PD-1 blockade in relapsed/refractory classical Hodgkin lymphoma, evidence supporting NLR as a predictive biomarker of response to nivolumab or pembrolizumab remains limited. Current studies suggest that composite inflammatory indices may outperform NLR alone in this setting. In DLBCL, high NLR predicts adverse outcomes following standard immunochemotherapy [51, 52] by R-CHOP and may also influence responses to CAR-T-cell therapy. Several CAR-T studies suggest that elevated pre-infusion NLR is associated with inferior expansion and persistence of CAR-T cells, although the NLR score has not been directly evaluated in these settings. Patients with lower inflammatory burden before lymphodepletion achieve superior durable responses following CD19 CAR-T therapy, demonstrating the need of strategies aiming at the optimization of immunotherapy [53]. In addition to the simple explanation that the prognostic value of NLR in immunotherapy reflects systemic inflammation, a biological rationale may support this observation. Mechanistically, elevated neutrophils may represent expansion of myeloid-derived suppressor cells (MDSCs), which inhibit T-cell activation through multiple mechanisms such as arginase-1, inducible nitric oxide synthase, and reactive oxygen species production as described by Gabrilovich and Nagaraj [54].

### Platelet-to-lymphocyte ratio

The platelet-to-lymphocyte ratio is defined as **PLR = Platelet Count / Absolute Lymphocyte Count.** Platelets actively contribute to cancer progression by secreting pro-inflammatory mediators such as platelet-derived growth factor (PDGF), VEGF, and TGF-β. In addition, platelets facilitate tumor cell survival and protect malignant cells from immune-mediated destruction. Emerging evidence suggests that elevated platelet activity contributes to T-cell exhaustion and reduced efficacy of immune checkpoint inhibitors through increased GARP-TGF-β signaling [55]. Elevated PLR has demonstrated prognostic significance in lymphoma [56, 57] where high PLR is associated with reduced PFS and OS. In multiple myeloma, the value of PLR ratio is debated since the study of Wongrakpanich et al. [58] showed a difference in median survival between high and low groups, i.e. 45 vs.62 months that did not reach statistical significance. In contrast, in the study by Solmaz et al. [59] applying a cutoff of 120.00, a decreased PLR showed a negative impact on the outcome. In these studies patients were mainly not treated by immunotherapy, so that their relevance regarding today treatment is low. Current evidence regarding the prognostic value of baseline PLR in CAR-T therapy is limited. Preliminary retrospective studies suggest that a higher baseline PLR may be associated with an increased risk of severe immune effector cell–associated toxicities [60], but evidence that lower baseline PLR predicts improved response or survival after CAR-T therapy is currently lacking. Prospective validation is required. Thus, the relevance of PLR in immunotherapy remains under investigation.

### Monocyte-to-lymphocyte ratio and lymphocyte-to-monocyte ratio

The monocyte-to-lymphocyte ratio is calculated as **MLR = Absolute Monocyte Count / Absolute Lymphocyte Count.** Monocytes are precursors of tumor-associated macrophages (TAMs), which are major mediators of immunosuppression within the tumor microenvironment. In lymphomas, elevated circulating monocyte counts have been repeatedly associated with inferior outcomes. The retrospective study of Wilcox et al. [61] included 366 patients with DLBCL. The authors demonstrated that an elevated absolute monocyte count (AMC) is associated with poorer survival, while a reduced absolute lymphocyte count (ALC) is also an adverse prognostic factor. Combining these two parameters into an Absolute Monocyte and Lymphocyte Prognostic Score (AMLPS) identifies a subgroup of high-risk patients, providing prognostic information that is independent of the IPI. High MLR may reflect increased recruitment of immunosuppressive macrophages capable of producing IL-10, TGF-β, and indoleamine 2,3-dioxygenase. In Hodgkin lymphoma, MLR has shown prognostic value comparable to established clinical scoring systems [62] in young but not in elderly (> 60 years) patients. Similar observations have been reported in CLL and DLBCL. Recent studies suggest that elevated MLR may identify patients with highly suppressive myeloid microenvironments who are less likely to benefit from T-cell-directed immunotherapies.

The lymphocyte-to-monocyte ratio represents the inverse relationship **LMR = Absolute Lymphocyte Count / Absolute Monocyte Count.** Low LMR reflects both impaired adaptive immunity and increased monocyte-driven immune suppression. Therefore, LMR emerged as a potential prognostic marker in Hodgkin lymphoma, DLBCL, follicular lymphoma, and peripheral T-cell lymphoma. Several meta-analyses have demonstrated that low LMR predicts inferior OS and PFS across lymphoid malignancies. Because lymphocytes are critical mediators of CAR-T and checkpoint inhibitor activity, LMR may indirectly estimate the capacity of patients to mount effective antitumor immune responses, both native and following immunotherapy. Emerging evidence indicates that LMR may outperform NLR in diseases where macrophage-rich microenvironments play a dominant biological role, particularly Hodgkin lymphoma. Nonetheless, in Hodgkin lymphoma the study of Porrata et al. [63] included 476 consecutive patients treated between 1974 and 2010, thus before the introduction of the recent immunotherapy treatment, limiting the usefulness of this score until clearly tested in the context of immunotherapy. Regarding DLBCL, the meta-analysis of Xia et al. [64] encompassing 12 studies and over 5,000 patients, confirmed that a low baseline LMR is significantly associated with poorer OS and PFS.

### Systemic immune-inflammation index and pan-immune-inflammation (PIV) and pan-immune-inflammation, lactate dehydrogenase, and ECOG performance status (PILE) index

The systemic immune-inflammation index integrates three cellular components, since **SII = Platelets × Neutrophils / Lymphocytes.** SII combines information from neutrophils, platelets, and lymphocytes into a single parameter and may therefore capture the complexity of systemic inflammation. Originally developed in hepatocellular carcinoma [65], SII has subsequently demonstrated prognostic value in multiple hematologic malignancies. In DLBCL, elevated SII predicts inferior survival independently of IPI and molecular subtype [66]. This result was confirmed by the meta-analysis of Fan et al. based on five articles including 592 cases: SII significantly predicted worse OS and PFS [67]. These results are relevant to immunotherapy since most patients were treated with chemotherapy associated with anti-CD20 monoclonal antibodies Mab. Similar findings have been reported for SII in multiple myeloma, although the study of Wang et al. [68] showed by a score associating NLR/LMR/PLR/IL-10 was more predictive. In non-M3 AML, the prognostic value of the SII has only recently been investigated [69]. In a retrospective cohort of patients with de novo non-M3 AML, a high baseline SII was significantly associated with inferior OS and EFS, and SII remained as an independent prognostic factor after adjustment for established clinical and laboratory variables, suggesting that it reflects the interplay between systemic inflammation and host immune status. Although these findings support the potential utility of SII as an inexpensive and readily available biomarker for risk stratification in AML, the current evidence is limited to a small number of retrospective studies and requires validation in large prospective and multicenter cohorts before routine clinical implementation.

The Pan-Immune-Inflammation Value (PIV) integrates neutrophil, platelet, monocyte, and lymphocyte counts and is calculated as (neutrophils × platelets × monocytes) / lymphocytes. By combining myeloid and lymphoid compartments, PIV reflects the balance between systemic inflammation and host immune status. The PILE score further integrates PIV with lactate dehydrogenase (LDH) and ECOG performance status, thereby incorporating systemic inflammation, disease burden/metabolic activity, and functional status. In hematologic malignancies, elevated PIV and PILE have been associated with inferior clinical outcomes. In particular, studies in primary central nervous system lymphoma and diffuse large B-cell lymphoma have reported associations with shorter overall and progression-free survival, while PILE was also associated with treatment response in primary CNS lymphoma [70, 71]. These findings support their potential prognostic relevance, although their predictive value for immunotherapy efficacy remains insufficiently validated.

### Prognostic nutritional index

The Prognostic Nutritional Index is calculated as **PNI = Albumin (g/L) + 5 × Absolute Lymphocyte Count (10⁹/L).** PNI reflects both nutritional status and immune competence. Hypoalbuminemia is a recognized marker of chronic inflammation mediated largely through IL-6-dependent suppression of hepatic albumin synthesis. Simultaneously, lymphopenia reflects impaired cellular immunity. Low PNI predicted poor outcomes in lymphoma [72] but there was no significance between survival and PNI in the multivariate analyses [73], since PNI was closely correlated with the well-established prognostic factors for DLBCL patients and was not an independent prognostic factor. In myeloma, two of the parameters used in the PNI are part of the Controlling Nutritional Status (CONUT) score, which high score was considered in the meta-analysis of Jin et al. [74] as related to a dismal OS but not to PFS, and independent of the ISS. In AML, a simplified CONUT score (eliminating total lymphocyte count) was more particularly interesting since it was independently associated with poor 2-year OS [75]. From an immunotherapy perspective, PNI may identify frail patients with reduced immune reserve and limited capacity for sustained T-cell activation. From a mechanistic perspective, hypoalbuminemia often reflects a pro-inflammatory state driven by cytokines such as Interleukin 6, Tumor Necrosis Factor alpha, and IL-1β, which promote tumor proliferation, immune evasion, and treatment resistance. Simultaneously, lymphopenia indicates reduced immune surveillance and diminished effector-cell availability, including CD4 + T cells, CD8 + cytotoxic lymphocytes, and natural killer cells. This immune dysfunction may compromise antigen recognition, cytokine secretion, and cytotoxic activity against malignant cells.

### Glasgow prognostic score and modified Glasgow prognostic score

The **Glasgow Prognostic Score combines C-reactive protein (CRP) and serum albumin**. The original GPS assigns a score of 0 for CRP ≤ 10 mg/L and albumin ≥ 35 g/L, a score of 1 to one abnormal value and 2 for both abnormal values. The modified GPS (mGPS) gives greater weight to CRP elevation. GPS and mGPS represent integrated measures of systemic inflammation driven largely by IL-6 signaling. Elevated CRP reflects activation of the innate immune system and correlates strongly with inflammatory cytokine production. Although initially developed in solid tumors [76], GPS and mGPS have shown prognostic significance in DLBCL, Hodgkin lymphoma, MM, and MDS. Because CRP is closely linked to cytokine-mediated immune dysfunction, GPS may be particularly relevant for predicting outcomes after immunotherapies associated with cytokine activation, including CAR-T-cell therapy. Recent studies have demonstrated associations between elevated baseline CRP and increased incidence of cytokine release syndrome (CRS), immune effector cell-associated neurotoxicity syndrome (ICANS), and reduced survival following CAR-T treatment [77].

**Table 2:** Major inflammatory prognostic scores used in hematologic malignancies

Score	Formula	Biological Interpretation	Main Hematologic Applications	Potential Relevance to Immunotherapy
NLR	Neutrophils / Lymphocytes	Myeloid inflammation vs. adaptive immunity	HL, DLBCL, AML, MM	CAR-T response, ICI outcomes
PLR	Platelets / Lymphocytes	Platelet-mediated inflammation	DLBCL, MM, AML	T-cell suppression, response prediction
MLR	Monocytes / Lymphocytes	Myeloid immunosuppression	HL, DLBCL, CLL	MDSC/TAM-rich microenvironment
LMR	Lymphocytes / Monocytes	Immune competence	HL, DLBCL, FL, PTCL	T-cell fitness estimation
SII	Platelets × Neutrophils / Lymphocytes	Global inflammatory burden	DLBCL, AML, MM	CAR-T efficacy prediction
PNI	Albumin + 5 × Lymphocytes	Nutritional and immune status	Lymphomas, AML, MM	Immune reserve and toxicity risk
GPS	CRP + Albumin	Systemic inflammation	DLBCL, HL, MM, MDS	CRS/ICANS risk, survival
mGPS	Modified CRP/Albumin score	IL-6-driven inflammation	DLBCL, MM, MDS	Cytokine-mediated toxicity prediction
PIV	Neutrophils x platelets x monocytes/lymphocytes	Inflammatory/immuno-suppressive host environment	DLBCL	Systemic immune-inflammatory markers
PILE	PIV + ecog Ps + LDH	Integrates systemic inflammation, tumor/metabolic burden and functional status	DLBCL	Diminished T-cell response and increased CRS/ICANS risk

Inflammatory prognostic scores offer several advantages as predictive biomarkers for immunotherapy: low cost and universal availability, easy longitudinal monitoring, reflection of both tumor and host immune biology, potential integration into composite predictive models. However, important limitations remain. Most evidence derives from retrospective studies. Cutoff values vary substantially among cohorts, and inflammatory markers may be influenced by infections, corticosteroids, chemotherapy, and comorbid conditions

## Endothelial activation scores: EASIX and modified EASIX (Table 3)

Beyond tumor-intrinsic and immune-related biomarkers, increasing attention has been directed toward the role of endothelial dysfunction in determining outcomes after immunotherapy. The vascular endothelium is now recognized as an active participant in immune regulation, cytokine trafficking, leukocyte migration, and inflammatory amplification. Endothelial activation contributes to systemic inflammation, coagulation abnormalities, capillary leak syndrome, and organ toxicity, all of which may influence the efficacy and safety of immune-based therapies. The endothelium constitutes a dynamic interface between immune cells and target tissues. Following CAR-T infusion or other potent immune interventions, massive cytokine release induces endothelial injury characterized by increased vascular permeability, activation of coagulation pathways, release of von Willebrand factor (vWF), angiopoietin-2 secretion, capillary leak syndrome and microvascular thrombosis. Experimental studies have shown that endothelial activation amplifies inflammatory cascades through reciprocal interactions with macrophages, monocytes, and activated T cells [78]. Several biomarkers have been associated with endothelial dysfunction during immunotherapy: Angiopoietin-2 (ANG2), vWF, soluble thrombomodulin, VEGF, ferritin, LDH. However, many of these biomarkers are not routinely available in clinical practice. EASIX was therefore developed as a simple surrogate score based on routinely measured laboratory parameters.

The original Endothelial Activation and Stress Index (EASIX) score is calculated as **LDH (U/L)×Creatinine (mg/dL) /Platelets (G/L).** The biological rationale for EASIX is closely linked to the endothelial dysfunction that characterizes severe immune effector cell toxicities. The EASIX score (LDH × creatinine / platelet count) integrates markers of tissue injury, renal dysfunction, and platelet consumption and can therefore serve as an indirect surrogate of systemic endotheliopathy. Following CAR-T-cell activation, inflammatory mediators including IL-1β, IL-6, IFN-γ and TNF-α activate and injure vascular endothelial cells, resulting in increased vascular permeability, disruption of endothelial junctions, platelet consumption and microvascular dysfunction. In the central nervous system, endothelial activation and pericyte dysfunction compromise blood-brain barrier (BBB) integrity, promoting leakage of plasma proteins, cytokines and immune cells into the CNS and contributing to cerebral edema and ICANS. Importantly, this process is not simply a consequence of systemic cytokine release but may become self-amplifying: endothelial and perivascular cells can respond to IL-1 and IL-6 by activating NF-κB and JAK/STAT pathways and producing additional inflammatory mediators, while IL-6 trans-signaling through soluble IL-6 receptor can further amplify endothelial activation and vascular permeability. This feed-forward interaction between cytokine signaling, endothelial injury and BBB disruption provides a biological basis for the association between elevated EASIX and severe CRS/ICANS and supports the use of EASIX as a surrogate marker of endothelial vulnerability in CAR-T-cell therapy [77, 78].

Higher EASIX values indicate greater endothelial stress and activation. The score is frequently log2-transformed in clinical studies to improve statistical modeling (log2 EASIX). The EASIX was initially developed as a surrogate marker of endothelial dysfunction in the allogeneic hematopoietic stem cell transplantation (allo-HSCT). The pretransplantation predictive utility of EASIX was demonstrated by Fujita [79] in patients undergoing allo-HSCT. In this study involving 317 patients, elevated pre-transplant EASIX independently predicted nonrelapse and overall mortality. These findings provided the biological rationale for exploring EASIX in cellular immunotherapy. To improve applicability in different clinical contexts, several modified versions of EASIX have been proposed. The most used modified score replaces creatinine with CRP, generating the **mEASIX = LDH (U/L) ×CRP (mg/L)/Platelet (G/L)**. The rationale is based on the major role of systemic inflammation in endothelial injury. Advantages of mEASIX include greater sensitivity to inflammatory activity, easier applicability in multicenter studies, and improved discrimination in some CAR-T cohorts.

In the study of Galli et al. [80] baseline mEASIX was predictive for consumptive coagulopathy and CRS graded 2 or higher, and for PFS and OS. Pennisi et al. [77] analyzed patients receiving CD19-directed CAR-T therapy and found that elevated pre-lymphodepletion EASIX was significantly associated with grade ≥ 3 CRS and inferior survival outcomes. The mEASIX measured before lymphodepletion predicted severe CRS and ICANS with performance comparable to more sophisticated cytokine panels. Despite encouraging results, several limitations remain. First, most studies are retrospective and involve relatively small cohorts. Second, cut-off values vary considerably across publications, preventing standardization. Third, EASIX may be influenced by factors unrelated to endothelial dysfunction, including renal impairment, hemolysis, infection, and advanced tumor burden. Fourth, prospective validation studies remain scarce, particularly outside CAR-T settings.

**Table 3:** Studies evaluating EASIX and mEASIX in immunotherapy

Study	Disease	Therapy	Endpoint	Main Findings
[79], 2023	Hematologic malignancies	Allo-HSCT	NRM, OS	High EASIX predicts endothelial complications and mortality
Gust et al. [78], 2017	B-cell malignancies	CAR-T	ICANS	Endothelial activation associated with neurotoxicity
Pennisi et al. [77], 2021	DLBCL, ALL	CD19 CAR-T	CRS, ICANS	Baseline EASIX predicts severe toxicity
Multiple retrospective cohorts (2021–2025)	NHL, ALL, MM	CAR-T	CRS/ICANS	Consistent association with toxicity severity
Emerging studies	Lymphoma, myeloma	Bispecific antibodies	Toxicity	Preliminary evidence; validation ongoing

There is growing evidence that EASIX and mEASIX are useful biomarkers of endothelial dysfunction and immunotherapy-related toxicity. Initially developed in the allo-HSCT setting, EASIX has consistently been associated with CRS and ICANS severity following CAR-T therapy across multiple retrospective cohorts. More recent studies suggest a potential role in predicting toxicity associated with bispecific antibodies, although prospective validation is still required

## CAR-T-specific prognostic scores: CAR-HEMATOTOX and modified CAR-HEMATOTOX (Table 4)

The introduction of CD19-directed CAR-T therapy has dramatically improved outcomes in relapsed or refractory B-cell malignancies. However, the efficacy of CAR-T cells is frequently accompanied by significant toxicities, including CRS, ICANS, prolonged cytopenia, severe infections, and non-relapse mortality (NRM). Among these complications, prolonged hematologic toxicity has emerged as one of the most clinically relevant adverse events because it significantly impacts quality of life, increases hospitalization rates, predisposes patients to infectious complications, and may compromise long-term survival. To address the need for risk stratification before CAR-T infusion, several prognostic tools have been developed. The most widely validated CAR-T-specific score is the **CAR-HEMATOTOX (HT) score**, which was specifically designed to predict severe hematologic toxicity following CAR-T-cell therapy. The CAR-HEMATOTOX score was initially developed by Rejeski and colleagues in a multicenter study involving patients with relapsed/refractory DLBCL treated with CD19-directed CAR-T therapy [81]. The rationale behind the score was based on the observation that prolonged cytopenias following CAR-T therapies are not solely attributable to lympho-depleting chemotherapy but are also influenced by pre-existing bone marrow reserve, systemic inflammation, and immune dysregulation before infusion. Baseline cytopenias and systemic inflammation may act synergistically to impair hematopoietic niche recovery after lymphodepletion and CAR-T-cell infusion. Pre-existing cytopenias reflect limited hematopoietic reserve and/or a compromised bone marrow microenvironment, including reduced availability and functional impairment of hematopoietic stem and progenitor cells. At the same time, elevated ferritin and C-reactive protein (CRP) identify a pro-inflammatory state that may promote cytokine-driven stromal and endothelial dysfunction, alter hematopoietic stem-cell trafficking and proliferation, and sustain inflammatory suppression of hematopoiesis. Following lymphodepletion, this reduced hematopoietic reserve is challenged by further marrow stress, while persistent inflammation may delay regeneration and increase susceptibility to prolonged cytopenias, infections, and transfusion requirements. Thus, CAR-HEMATOTOX integrates both **hematopoietic reserve** and **inflammatory burden**, providing a biologically plausible composite measure of the capacity of the bone marrow niche to recover after CAR-T therapy [81, 82].

The score combines five routinely available laboratory parameters measured before lymphodepletion: absolute neutrophil count (ANC), hemoglobin level, platelet count, CRP, ferritin. Low value for ANC, hemoglobin and platelets counts for 1 point each, while 1 point is attributed to elevated CRP and ferritin. In its original application, patients were primarily dichotomized into low-risk (score 0–1) and high-risk (score ≥ 2) groups. In subsequent analyses, higher scores have also been further stratified into intermediate-to-high-risk (2–4) and ultra-high-risk (≥ 5) categories. These variables reflect two major biological domains, i.e. baseline hematopoietic reserve and pre-existing inflammatory activation. Patients classified as HT-high exhibit substantially increased risks of prolonged neutropenia, severe thrombocytopenia, severe anemia, febrile neutropenia, infectious complications and intensive care admission [81]. In the CD19 CAR-T therapy for acute lymphoblastic leukemia (ALL), CAR-HEMATOTOX low scores had lower rates of delayed neutrophil recovery than those with HThigh scores, fewer severe infections, higher response rates, higher OS, higher EFS, as well as lower NRM and cumulative incidence of relapse. The survival differences remained significant after multivariable adjustment for disease burden and other covariates [83]. In MM BCMA-directed CAR-T therapy, HThigh patients displayed prolonged severe neutropenia, increased severe infection rate, more severe ICANS, higher one-year NRM (predominantly attributable to fatal infections) [84]. Response rates according to IMWG criteria were higher in HTlow patients, and HThigh patients exhibited inferior PFS and OS [84].

Although the original CAR-HEMATOTOX score demonstrated robust performance in DLBCL, investigators recognized potential limitations when applying it to other hematologic malignancies and CAR-T platforms. Several groups therefore developed modified versions of the score. The modified CAR-HEMATOTOX (mHT) score generally preserves the original biological concept while adapting variable weighting or cutoff values according to specific patient populations. Published modifications have sought to improve discrimination of severe cytopenia, enhance prediction of infectious events, adapt the score to MM CAR-T therapy (idecabtagene vicleucel, ciltacabtagene autoleucel) and facilitate application in real-world cohorts [85].

**Table 4:** Summary of CAR-T-specific prognostic scores

Score	Variables Included	Primary Endpoint	Main Findings	Clinical Utility
CAR-HEMATOTOX	ANC, hemoglobin, platelet count, CRP, ferritin	Prolonged hematologic toxicity	Predicts severe neutropenia, thrombocytopenia, infections, NRM	Pre-CAR-T risk stratification
Modified CAR-HEMATOTOX (mHT)	Adapted versions of HT variables with disease-specific cutoffs	Cytopenias and infectious complications	Improved applicability in real-world cohorts and alternative CAR-T platforms	Expanded use across lymphoma and myeloma

In CAR-T-specific prognostic approaches, with CAR-HEMATOTOX integrating systemic inflammation and bone marrow reserve, a high-risk profile identifies patients at increased risk of prolonged cytopenias, infections, toxicity, and poorer survival outcomes. Modified versions extend its applicability across different diseases and CAR-T platforms, supporting its use for pre-infusion risk stratification and monitoring

## Emerging prognostic scores and integrated biomarker models (Table 5)

### T-cell fitness

Gene-expression profiling has identified immune programs associated with immunotherapy response. Examples include response-associated signatures such as Interferon-gamma pathway activation, cytotoxic T-cell activation, antigen presentation pathways, together with resistance-associated signatures such as T-cell exhaustion, MYC activation, metabolic dysfunction and immunosuppressive myeloid programs. Single-cell RNA sequencing (scRNA-seq) has enabled identification of cellular ecosystems predictive of immunotherapy efficacy, so that emerging composite scores integrate analysis of T-cell subsets, NK-cell subsets, myeloid populations, exhaustion markers, cytokine signaling pathways, this approach providing substantially greater resolution than conventional flow cytometry. The biological validity of T-cell fitness assessment is supported by the central role of T-cell differentiation, persistence, metabolic competence, and exhaustion in determining the activity of cellular and immune-based therapies. However, translation into clinically actionable biomarkers remains challenging because studies use heterogeneous marker combinations, thresholds, sampling times, and analytical platforms. Conventional flow cytometry remains relatively accessible and moderately costly, whereas high-dimensional flow cytometry, mass cytometry, transcriptomic profiling, and scRNA-seq require specialized expertise, instrumentation, and bioinformatics. Their clinical implementation therefore requires standardized panels, validated thresholds, reproducible analytical pipelines, and demonstration of incremental clinical value over established prognostic variables.

One of the most important recent concepts in immunotherapy is the notion of “immune fitness”. Studies have demonstrated that baseline characteristics of circulating T cells strongly influence outcomes after CAR-T therapy, bispecific antibodies or checkpoint blockade. Several investigators have developed composite scores based on favorable features [high proportion of stem-cell memory T cells (TSCM), high central-memory T cells, preserved CD27 expression, and preserved CD28 expression] and unfavorable features (high PD-1 expression, high TIGIT expression, high LAG-3 expression, high TIM-3 expression, and terminal exhaustion signatures). Patients exhibiting an immune-fitness phenotype demonstrate better CAR-T expansion, longer CAR persistence, higher complete response rates and improved long-term survival. The GRIm (Gustave Roussy Immune Score) score was based on albumin, LDH and NLR score, each established prognostic biomarkers in lymphoma, MM, and CAR-T settings, supporting the biological relevance of this composite score although it was initially developed with solid cancers and has not been firmly validated in hematological malignancies [86]. The Combined Immune Score (CIS) in MM combines the LMR score to the non-involved immunoglobulin level and in a multivariate analysis was a strong predictor of poor treatment-free survival (TSS) [87]. The hypothesis of the study of Bruins et al. [88] is that the calendar age of patients does not reflect an individual’s immune status. This score requires a sophisticated analysis of T-cell subpopulations and can be synthesized as immune age = (senescent + exhausted T cells) / (naive + recent thymic emigrants [RTE]). Differences between immune age and calendar age ranged from − 26 to + 23 years, with 35% of patients having a gap of ≥ ± 10 years. The authors concluded that the calendar age was not predictive of survival, in contrast to the immune age that was associated with PFS and OS [88], this significance being maintained even after adjusting for World Health Organization (WHO) performance status, ISS stage and International Myeloma Working Group **(**IMWG) frailty status [88].

### NK-cell competency

NK-cell competency is increasingly recognized as a relevant determinant of response to immunotherapy, particularly because NK cells represent major mediators of antibody-dependent cellular cytotoxicity (ADCC), a mechanism underlying the activity of several monoclonal antibody-based therapies. Beyond their role in ADCC, accumulating evidence indicates that NK cells are directly regulated by immune checkpoint pathways and may contribute to the clinical efficacy of immune checkpoint inhibitors (ICIs). Indeed, classical T-cell checkpoints, such as PD-1, are also expressed by a subset of NK cells [89–92], and PD-1 engagement by PD-L1 expressed on tumor cells can impair NK-cell cytotoxic functions [90, 93]. Conversely, PD-1/PD-L1 blockade may restore NK-cell effector activity, highlighting NK cells as potential contributors to the response to anti-PD-1/PD-L1 therapies rather than merely indirect biomarkers of T-cell immunity. Among NK-cell-specific inhibitory pathways, the CD94/NKG2A-HLA-E axis has emerged as a major immune checkpoint. NKG2A is highly expressed on NK cells and can also be induced on a fraction of tumor-infiltrating cytotoxic T cells, where it contributes to immune escape through recognition of HLA-E expressed by tumor cells [93]. Clinical and preclinical studies have shown that NKG2A blockade, alone or in combination with PD-1/PD-L1 inhibition, can enhance NK-cell degranulation, cytokine production and anti-tumor activity, supporting the development of combination checkpoint strategies targeting both adaptive and innate immune resistance mechanisms. NKG2A blockade with monalizumab is currently being explored in clinical trials across solid and hematological malignancies [94–96], including settings where NK-cell activity represents a relevant therapeutic component. Consequently, emerging NK-cell-based biomarkers extend beyond quantitative parameters (NK-cell counts, CD56bright/CD56dim distribution) and include functional and inhibitory receptor profiles. Candidate markers under investigation include activating receptors (NKG2D, NKp30, NKp46, NKG2C), CD16-mediated cytotoxicity, degranulation capacity (CD107a/LAMP1 expression), perforin and granzyme content, IFN-γ production, as well as exhaustion/senescence-associated receptors such as NKG2A, TIGIT, KLRG1 and CD57 [97, 98]. Integrated evaluation of these parameters may provide a more accurate representation of NK-cell fitness and predict responsiveness to immunotherapeutic approaches. In DLBCL, a prognostic signature based on the expression of seven NK-cell-related genes (MAP2K1, PRKCB, TNFRSF10B, IL-18, LAMP1, RASGRP1 and SP110) identified patient groups with significantly different overall survival, although validation in prospective cohorts is still required [99]. In MM, expansion of CD16+/CD226low NK-cell subsets characterized by impaired adhesion and reduced effector functions has been associated with poor survival [100]. In AML, the Natural Cytotoxicity Receptor (NCR)dull NK-cell phenotype correlates with adverse prognosis, while recovery of NCR expression is mainly observed in patients achieving CR, suggesting potential prognostic value both before and after treatment [101, 102]. However, NK-cell parameters have not yet been incorporated into formal risk stratification systems such as WHO, European LeukemiaNet or International Consensus classifications in AML, which remain largely based on genetic and cytogenetic abnormalities [103]. The biological validity of NK-cell profiling is particularly relevant when the therapeutic mechanism directly depends on NK-cell activity, as with ADCC-inducing monoclonal antibodies and approaches targeting NK-cell checkpoints. Functional parameters such as degranulation, target-cell killing, serial killing capacity, and cytokine production may provide information complementary to NK-cell enumeration or receptor expression. Nevertheless, NK-cell phenotypes are dynamic and influenced by disease burden, previous treatment, infections, cytokine exposure, and sample handling. Conventional NK-cell immunophenotyping by multiparameter flow cytometry is relatively accessible in specialized hematology laboratories, whereas functional assays, high-dimensional profiling, and single-cell approaches require greater technical expertise and resources. Their clinical adoption will require reproducibility, clinically validated thresholds, and evidence of added predictive value.

### Cytokine profiling

Cytokines provide a dynamic representation of the host immune response and are increasingly incorporated into predictive algorithms. The post-perfusion elevation of IL-6, IL-10, IFN-γ, MCP-1, GM-CSF and TNF-α has been associated with severe CRS, severe ICANS and treatment failure. Composite cytokine signatures are increasingly replacing single biomarkers. Examples include the pro-inflammatory signature IL-6/IL-8/TNF-α/CRP and the Immunosuppressive signatu**re** IL-10/TGF-β/soluble PD-L1. These signatures may identify patients with primary resistance, early relapses and excessive toxicity, although they do not have been formally standardized as a defined score. The biological rationale for cytokine profiling is strong because circulating cytokines provide a dynamic readout of immune activation, inflammation, myeloid-cell activity, and systemic toxicity. However, cytokine concentrations are highly sensitive to pre-analytical and analytical variables, including sampling time, anticoagulant, processing delay, storage conditions, assay platform, and detection limits. Single-cytokine measurements such as IL-6 are relatively accessible, whereas multiplex platforms capable of quantifying multiple cytokines require specialized instrumentation and are more expensive. Multiplex profiling nevertheless allows coordinated assessment of inflammatory and immunosuppressive pathways from limited sample volumes. Standardization of sampling procedures, analytical platforms, reference ranges, and clinically validated thresholds remains necessary before these signatures can be routinely used.

### Prognostic versus predictive value

Taken together, T-cell fitness, NK-cell competency, and cytokine profiling provide complementary dimensions of the host immune state, but their prognostic and predictive implications should be clearly distinguished. A prognostic biomarker provides information about clinical outcome irrespective of the treatment received, whereas a predictive biomarker identifies patients who are more or less likely to derive benefit from a specific therapeutic intervention. Several of the parameters described above currently have predominantly prognostic evidence; an association with PFS or OS does not by itself demonstrate treatment-specific predictive value. Conversely, parameters directly related to the mechanism of action of a therapy, such as T-cell proliferative and persistence capacity in CAR-T therapy, CD16-dependent NK-cell function during antibody-based therapy, or cytokine dynamics during immune-effector-cell therapy, may have greater predictive potential, but this requires demonstration of differential treatment effects according to biomarker status.

**Table 5:** Emerging prognostic scores and biomarker models in hematologic immunotherapy

Score / Model	Components	Main Setting	Predicted Outcomes	Current Level of Evidence	Refs.
T-cell Fitness Score	TSCM, memory T cells, exhaustion markers	CAR-T, bispecific antibodies	Response and persistence	Moderate	[104]
NK-cell Functional Score	NK phenotype and cytotoxicity markers	Chemotherapy	Response prediction	Emerging	[105]
Cytokine Risk Score	IL-6, IL-10, IFN-γ, TNF-α	CAR-T	CRS and ICANS	Moderate	[106]
Immune Activation Signature	Multiplex cytokines	CAR-T, ICI	Response and toxicity	Emerging	[78]
Transcriptomic Score	Gene-expression signatures	Multiple diseases	Response prediction	Emerging	[107]
Single-cell Immune profiling	scRNA-seq immune ecosystem analysis	AML	Response and resistance	Emerging	[108]
AI-based Composite Models	Clinical + biological + molecular variables	Multiple immunotherapies	Response, toxicity, survival	Emerging	[109]
ctDNA-MRD Integrated Score	ctDNA kinetics + MRD	CAR-T, bispecific antibodies	Relapse prediction	Emerging	[110]
Functional Immune Assays	Cellular activation and dynamics	Future immunotherapy platforms	Functional response prediction	Experimental	Théodoly/ Robert/ Delhaye, WO2023222842A1 EP4526428A1

In emerging biological and multimodal models, T-cell and NK-cell fitness, cytokine profiles, transcriptomic signatures, and single-cell analyses provide complementary information on immune competence, response, and resistance. Although most approaches remain exploratory, their integration with ctDNA/MRD, functional assays, and AI-based models may enable more precise and individualized prediction of response, toxicity, and survival

Finally, functional immune assays are perhaps the real next generation of prognostic scoring, since *de facto* they integrate all previous items of prognostic score as the ultimate response to effector cells to immunotherapy and in addition tumor cell sensibility to immune response. An especially innovative field concerns quantitative functional assessment of immune cells. An interesting approach has been developed by Sarkar et al. [111] in MM using a digital microfluidic bioassay platform, in which single cell assays are performed in microdroplets that serve as microreactors. Using this platform, the authors developed a multidimensional approach allowing to test the basic cytotoxic properties of NK cells but also, their effective killing of tumoral plasma cell and the effect of various immunotherapy such as lenalidomide (supposed to increase NK killing) and blockade of the PD1/PDL1 axis. The authors also developed an agent-based mathematical model to anticipate the outcome of treatment towards personalized medicine. A key challenge is to adapt these sophisticated methods so that they can be integrated into routine clinical practice as tools to support immunotherapy. This requires methods that are cost-effective, involve minimal hands-on time, provide rapid turnaround times, and require only small sample volumes.

### Micro imprinting of antibodies and biomolecules for cell phenotyping and activation

**Fig. 1:**
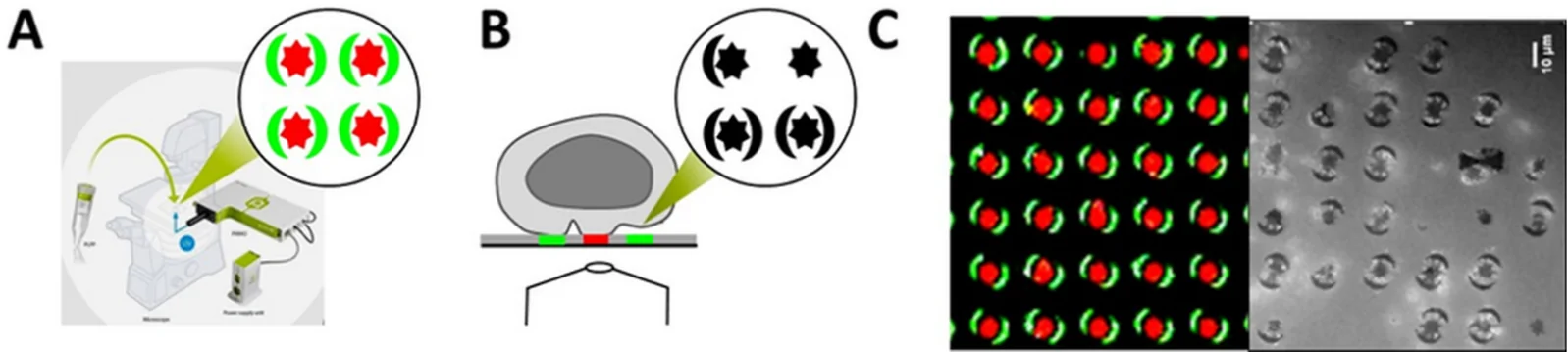
Example of microarray to assess leukocytes functions without immunostaining. **A**: The printing process by Light-Induced Molecular Adsorption of Proteins (LIMAP) allows multiple printing of distinct antibody patterns. This is an example of an array whose patterns hosting single cells are composed of two different zones (red and green). **B**: Considering a device with red patterns to capture and trigger activation of a leukocyte of interest (e.g. T cells with anti-CD3 and anti-CD28), the green patterns reveals activation (e.g. anti-CD69, anti-CD25, anti-CD107a), while interference microscopy reveals the fingerprint of cell adhesion (dark = adhesion, bright non adherent). A non-activated T cell displays a dark central star, whereas activated T cell display also dark moon quarters. (**C**) Fluorescence microscopic picture of a double-pattern microarray of aCD3/aCD28 (red) and aCD69 (green) on left, interferometric images of T cells after 3 h on the microarray on the right, showing activated cells adhering on both central and surrounding patterns

Recently was developed a technology based on microfluidic and micro patterning that allows single cell functional assays on custom microarrays without immunostaining [112]. The method uses optical microscopy and surface chemistry processes to realize multiple micropatterns of different proteins at subcellular size (Fig. 1). Some of the authors contributing to the development of this approach, including Philippe Robert and Olivier Théodoly, are inventors on intellectual-property applications related to the technology (EP4526428A1 and PCT/EP2023/063407). This constitutes a relevant potential conflict of interest and is explicitly disclosed here. The authors’ involvement in the development of the technology does not, in itself, establish bias in the reported observations ; nevertheless, independent validation in external cohorts and by investigators without intellectual-property interests will be important to establish the analytical and clinical utility of the platform measurement of immune-cell activation, adhesion, migration, and signaling at the single-cell level.

Depending on the pattern designs and their protein composition, the micro-arrays may be designed to perform various functions on immune cells without technician handling nor immunostaining, such as (1) selection/identification of a cell type of interest (2), triggering of an activation signal, and (3) read-out of the immune activation kinetics (4), cellular cytotoxicity against tumor cell lines or autologous tumor cells when available (5) quantification of T and NK-cell migration velocity and inter-target interval. These approaches generate dynamic parameters rather than static biomarkers, together with single cell information and multiplexed read-outs. Such a platform may form the basis of functional immunotherapy response scores, and a start-up (https://biomedical.mohican-technologies.com/) is developing this approach to eventually propose automatic and multiplexed micro assays for hospital routine practice. Potential parameters include activation kinetics, synapse formation efficiency, migration velocity, cytotoxic interaction dynamics and mechanical responsiveness. Unlike conventional biomarkers, these measurements evaluate the actual functional competence of immune effector cells. Such approaches align with the growing concept that “immune function” may be more predictive than simple immune cell counts. We propose to evaluate a novel integrative prognostic score (see Supplementary data) corresponding to the data collected via the micro assay method, integrating an Activation Kinetic Score (AKS), an Immune Synapse Efficiency Score (ISES), a Migration Competence Score (MCS), a Serial Killing Score (SKS), an ADCC Functional Score (AFS) and finally a Tumor Susceptibility Index (TSI). Nonetheless, at the hypothesis stage and in absence of robustly validated data, we cannot assign a weight for each category. The supplementary data section corresponds to an unvalidated conceptual framework rather than an established clinical score. The weight of each parameter should be modified by Machine Learning evaluation of clinical relevance of each parameter.

## Conclusion

Accumulating evidence highlights the prognostic and predictive potential of baseline clinical and biological biomarkers across a rapidly expanding spectrum of cancer immunotherapies. Composite indices integrating systemic inflammation, immune competence, nutritional status, endothelial dysfunction, and hematopoietic reserve, including NLR, LMR, PLR, PNI, and EASIX, have emerged as readily available, minimally invasive, and cost-effective tools for patient stratification. Rather than representing isolated parameters, these biomarkers capture the complex interplay between host immunity, tumor-driven inflammation, tissue homeostasis, and treatment-related vulnerability, thereby providing a surrogate measure of the biological fitness of the patient receiving immunotherapy. However, despite their promising clinical relevance, widespread implementation remains constrained by substantial heterogeneity among studies, including differences in tumor entities, therapeutic modalities, patient populations, analytical methodologies, and biomarker thresholds. Future efforts should therefore focus on prospective multicenter validation, harmonization of scoring systems, and the development of dynamic models integrating conventional laboratory parameters with high-dimensional immune profiling, transcriptomic, spatial, and functional approaches. Ultimately, moving beyond static prognostic markers toward a comprehensive understanding of host immune fitness and tumor–immune ecosystem interactions will be essential to enable a truly personalized approach to cancer immunotherapy. The integration of multidimensional biomarkers may allow more accurate patient selection, early identification of resistance or toxicity risk, optimization of therapeutic strategies, and, ultimately, improvement of long-term clinical outcomes.

## Electronic Supplementary Material

Below is the link to the electronic supplementary material.

Supplementary Material 1

## Data Availability

No datasets were generated or analysed during the current study.

## Abbreviations

ADCC: Antibody-dependent cellular cytotoxicity
AFS: ADCC Functional Score
AKS: Activation Kinetics Score
ALL: Acute lymphoblastic leukemia
AML: Acute myeloid leukemia
AMLPS: Absolute Monocyte and Lymphocyte Prognostic Score
ANG2: Angiopoietin-2
BCMA: B-cell maturation antigen
CAR: Chimeric antigen receptor
CAR-T: Chimeric antigen receptor T-cell
CLL: Chronic lymphocytic leukemia
CLL-IPI: Chronic Lymphocytic Leukemia International Prognostic Index
CONUT: Controlling Nutritional Status
CR: Complete remission
CRP: C-reactive protein
CRS: Cytokine release syndrome
ctDNA: Circulating tumor DNA
DLBCL: Diffuse large B-cell lymphoma
EASIX: Endothelial Activation and Stress Index
ECOG: Eastern Cooperative Oncology Group
EFS: Event-free survival
ELN: European LeukemiaNet
FL: Follicular lymphoma
FLIPI: Follicular Lymphoma International Prognostic Index
GPS: Glasgow Prognostic Score
GRIm: Gustave Roussy Immune Score
Hb: Hemoglobin
HL: Hodgkin lymphoma
ICANS: Immune effector cell-associated neurotoxicity syndrome
ICI: Immune checkpoint inhibitor
IFN-γ: Interferon-gamma
IGHV: Immunoglobulin heavy-chain variable region
IL: Interleukin
IPI: International Prognostic Index
IPSS: International Prognostic Scoring System
IPSS-M: Molecular International Prognostic Scoring System
IPSS-R: Revised International Prognostic Scoring System
IPS: International Prognostic Score
ISES: Immune Synapse Efficiency Score
LDH: Lactate dehydrogenase
LMR: Lymphocyte-to-monocyte ratio
MCS: Migration Competence Score
MDS: Myelodysplastic syndromes
MDSC: Myeloid-derived suppressor cell
mGPS: Modified Glasgow Prognostic Score
MLR: Monocyte-to-lymphocyte ratio
MM: Multiple myeloma
MRD: Measurable residual disease
mHT: Modified CAR-HEMATOTOX
NCR: Natural cytotoxicity receptor
NK: Natural killer
NLR: Neutrophil-to-lymphocyte ratio
NCCN-IPI: National Comprehensive Cancer Network International Prognostic Index
NRM: Non-relapse mortality
OS: Overall survival
PD-1: Programmed cell death protein 1
PD-L1: Programmed death-ligand 1
PFS: Progression-free survival
PILE: PIV + LDH + ECOG PS
PNI: Prognostic Nutritional Index
PLR: Platelet-to-lymphocyte ratio
PIV: Pan-immune-inflammation value
PS: Performance status
PTCL: Peripheral T-cell lymphoma
R-CHOP: Rituximab, cyclophosphamide, doxorubicin, vincristine and prednisone
R-IPI: Revised International Prognostic Index
R-ISS: Revised International Staging System
R2-ISS: Second Revision of the International Staging System
scRNA-seq: Single-cell RNA sequencing
SII: Systemic immune-inflammation index
SKS: Serial Killing Score
TAM: Tumor-associated macrophage
TGF-β: Transforming growth factor-beta
TIGIT: T-cell immunoreceptor with Ig and ITIM domains
TIM-3: T-cell immunoglobulin and mucin-domain containing-3
TNF-α: Tumor necrosis factor-alpha
TSCM: Stem cell memory T cells
TSI: Tumor Susceptibility Index
VEGF: Vascular endothelial growth factor
vWF: Von Willebrand factor
