# Supplementary material for: Prognostic scores and biomarkers in hematologic malignancies: current evidence and future perspectives
Source: Biomark Res. 2026 Oct 31;14:157. doi: 10.1186/s40364-026-01033-8 (PMC13723931; doi:10.1186/s40364-026-01033-8)
Supplement: Supplementary file 1 [file 40364_2026_1033_MOESM1_ESM.docx]

**Supplementary Data**

The cutoff values are merely arbitrary values intended to serve as examples. The grouping of values is currently based only on the range of variability in the observed values—which roughly corresponds to a quartile distribution. These data correspond to an unvalidated conceptual framework rather than an established clinical score, This is why question marks were introduced into the tables.

**Sup. Data 1. FICS score**

**a. Activation Kinetics Score (AKS):** Measured after stimulation on activating micropatterns. Parameters: Time to first calcium flux, Time to CD69 upregulation, Time to granule polarization Scoring:

| **Activation Delay** | **Points** |
| --- | --- |
| <5 min | ? |
| 5–15 min | ? |
| 15–30 min | ? |
| >30 min | ? |

**Rationale:** Rapid activation reflects preserved immune responsiveness and reduced exhaustion.

**b. Immune Synapse Efficiency Score (ISES):** Measured on activating microchips. Parameters: Percentage of stable synapses, Synapse duration, Lytic granule polarization index. Scoring:

| **Stable Synapse Formation** | **Points** |
| --- | --- |
| >80% ? |  |
| 60–80% ? |  |
| 40–60% ? |  |
| <40% ? |  |

**Rationale:** Efficient synapse formation is required for cytotoxic activity.

**c. Migration Competence Score (MCS):** Measured in microfluidic migration chambers. Parameters: NK-cell migration velocity, Directionality index, Fraction of motile cells. Scoring:

| **Velocity** | **Points** |
| --- | --- |
| >12 μm/min | ? |
| 8–12 μm/min | ? |
| 4–8 μm/min | ? |
| <4 μm/min | ? |

**Rationale:** Tumor infiltration is critically dependent upon migration capacity.

**d. Serial Killing Score (SKS):** Measured in double-cell microchips. Parameters: Number of tumor cells killed per NK cell, Inter-target interval. Scoring:

| **Kills/NK Cell** | **Points** |
| --- | --- |
| ≥5 | ? |
| 3–4 | ? |
| 1–2 | ? |
| 0 | ? |

**e. Inter-target interval modifier:**

<20 min : +1 point, >60 min : −1 point

**Rationale:** Serial killing is one of the strongest indicators of antitumor immune potency.

**f. ADCC Functional Score (AFS):** Measured using tumor cells incubated with therapeutic antibodies. Examples: Rituximab, Obinutuzumab, Daratumumab, Elotuzumab. Parameters: Percentage of tumor lysis, Killing kinetics. Scoring:

| **Tumor Lysis** | **Points** |
| --- | --- |
| >75% | ? |
| 50–75% | ? |
| 25–50% | ? |
| <25% | ? |

**Rationale:** Directly evaluates the capacity of immune cells to cooperate with therapeutic antibodies.

**g. Tumor Susceptibility Index (TSI):** Unique feature of the score. Measured using autologous tumor cells whenever available. Parameters: Time-to-kill, Resistance to serial attacks, Recovery after immune contact Scoring:

| **Susceptibility** | **Points** |
| --- | --- |
| High | ? |
| Intermediate | ? |
| Low | ? |
| Resistant | ? |

**Rationale:** Response to immunotherapy depends not only on immune-cell competence but also on tumor-cell vulnerability.

**h. Final Score**

**FICS = AKS + ISES + MCS + SKS + AFS + TSI:**

Range: 0–18 points

**Risk Groups**

| **FICS Score** | **Interpretation** |
| --- | --- |
| ?– ? | Excellent functional immune competence |
| ?– ? | Good functional competence |
| ?– ? | Intermediate competence |
| ?– ? | Severely impaired immune function |

**Sup. Data 2. FICS 2.0 score**

A future version could integrate: FICS 2.0 = ?(Functional Score) + **?**(Immune Fitness) +**?**(Inflammatory Score) + **?**(Tumor Burden) where:

- Functional Score = microfluidic assay output
- Immune Fitness = T-cell/NK-cell phenotyping
- Inflammatory Score = NLR, PNI, mGPS
- Tumor Burden = LDH, ctDNA, PET parameters

Univariate logistic regression is used to assess the association of each candidate predictor with prognosis / treatment efficiency; candidates with a significance level of 0.05 are selected. Then, multivariable logistic regression is used to identify independent predictors of prognosis / treatment efficiency with an alpha risk of 0.05. In the same way, cox regression method can be used for survival analyses. Furthermore, chosen variables (Xi) are assigned a weighted integer (biXi), based on the multivariate model coefficient (and its standard error). Finally, the integers are added together to calculate the total risk score. The discriminatory performance of the score is assessed using the receiver operating curve (ROC) method. S=
